# Hydration status and physiological workload of UAE construction workers: A prospective longitudinal observational study

**DOI:** 10.1186/1745-6673-3-21

**Published:** 2008-09-18

**Authors:** Graham P Bates, John Schneider

**Affiliations:** 1 School Public Health, Curtin University, Perth, Australia; 2 Department of Community Medicine, Faculty Medicine and Health Sciences, UAE University, Al Ain, United Arab Emirates

## Abstract

**Background:**

The objective of the study was to investigate the physiological responses of construction workers labouring in thermally stressful environments in the UAE using Thermal Work Limit (TWL) as a method of environmental risk assessment.

**Methods:**

The study was undertaken in May 2006. Aural temperature, fluid intake, and urine specific gravity were recorded and continuous heart rate monitoring was used to assess fatigue. Subjects were monitored over 3 consecutive shifts. TWL and WBGT were used to assess the thermal stress.

**Results:**

Most subjects commenced work euhydrated and maintained this status over a 12-hour shift. The average fluid intake was 5.44 L. There were no changes in core temperature or average heart rate between day 1 and day 3, nor between shift start and finish, despite substantial changes in thermal stress. The results obtained indicated that the workers were not physiologically challenged despite fluctuating harsh environmental conditions. Core body temperatures were not elevated suggesting satisfactory thermoregulation.

**Conclusion:**

The data demonstrate that people can work, without adverse physiological effects, in hot conditions if they are provided with the appropriate fluids and are allowed to self-pace. The findings suggested that workers will self-pace according to the conditions. The data also demonstrated that the use of WBGT (a widely used risk assessment tool) as a thermal index is inappropriate for use in Gulf conditions, however TWL was found to be a valuable tool in assessing thermal stress.

## Background

The United Arab Emirates and other Gulf States have thousands of expatriate workers performing physical tasks in very hostile environmental conditions during summer. To date there have been few studies to document the hydration status and possible fatigue of these workers whilst working in the heat. The environmental conditions in the summer are some of harshest in the world. As a consequence it is frequently proposed that it is beyond the physiological thresholds of these workers to work safely, however, little data has been gathered to better understand the physical strain imposed on these workers. In addition the hydration status of these workers has not been documented.

Maintaining a stable core body temperature in the face of changing environmental conditions and metabolic workloads allows humans to function in diverse climates and surroundings. In hot conditions, thermoregulation depends upon the dissipation of body heat to the environment. Sweating cools the skin by evaporation and is the principal heat loss mechanism when working in very hot environments. Increased blood flow to the periphery of the body can also cause significant heat loss through convective currents and radiation.

### Hydration

The rate of perspiration varies considerably, depending upon the climatic conditions, exercise intensity and clothing worn [1]. Sweat rates between 0.3 and 1.5 L per hr can be expected of workers in hot climates [2], resulting in large volumes of fluid loss over the course of a day. This can result in dehydration if adequate fluid replacement does not occur. In thermally stressful conditions such as occur in the UAE during summer, structured rehydration maybe required, as discretionary fluid consumption to avoid thirst may not be adequate to prevent dehydration. Drinking at mealtimes is important because eating encourages fluid intake, and electrolytes in food promote water absorption as well as replacing sweat losses [3].

The major short-term implications of dehydration are the result of a depleted blood volume and the consequent cardiovascular strain. Sweat is hypotonic to blood and causes water loss from both the intracellular and extracellular compartments, with most significant effects occurring due to plasma depletion. The reduced blood volume causes a compensatory increase in heart rate of around 10 beats.min^-1 ^for every one percent of body weight lost [4]. Heat causes additional cardiovascular strain because blood is required for heat loss as well as maintaining adequate perfusion to working muscles. Thus evaporative and convective heat loss become less efficient when an individual is dehydrated, as sweating [5] and skin blood flow [6] are both reduced. Consequentially, core temperature rises, with increases occurring at 1% hypohydration. Core temperature continues to rise as dehydration progresses, with no advantage being conferred by acclimatisation [7,8]. Core body temperature increases at a greater rate in hypohydrated subjects, and at the same time, they exhibit reduced tolerance to elevated temperature [9].

Studies have shown that core body temperature, heart rate and cardiac output reach certain critical values at the point of exhaustion [10]. Thus it follows that dehydration, which elevates both heart rate and core temperature, causes significant physical performance decrements. Water deficits of 1–2% of body weight in a moderate environment results in a 6–7% reduction in physical work capacity, water loss of 3–4% of body weight in the same environment causes a reduction of 22% physical work capacity [11]. The additional cardiovascular strain imposed by a hot environment means that a 4% body water loss can cause a physical work capacity reduction of around 50% [12]. Other factors associated with dehydration that accelerate fatigue are increased rate of glycogen depletion, greater metabolite accumulation and decreased psychological drive for work or exercise [13].

Dehydration also has marked cognitive effects. Performance in intellectual tests is affected at 2% hypohydration, and becomes progressively worse as water deficit increases [14]. Impaired concentration, reasoning and mood can occur due to dehydration and the concomitant increase in core body temperature. Not surprisingly, workplace accidents are more common in hot environments, and are often associated with heat stress and dehydration [15].

More deleterious health effects can occur if dehydration is allowed to progress, as it increases the likelihood of heat related illness. A number of conditions are associated with heat stress and dehydration, namely heat rash, heat exhaustion, heat cramps, heat oedema, heat syncope (fainting), and chronic heat fatigue. Thermoregulatory failure can occur in severe cases of dehydration and hyperthermia, resulting in heat stroke, an often fatal condition [16].

Several long-term health consequences of dehydration have been documented. There is a well-known link between inadequate fluid intake and renal calculi (kidney stones), and a recent study illustrated a high incidence of bladder cancer in subjects who had experienced chronic dehydration [17].

It is therefore imperative that workers performing physical work in hot conditions maintain their hydration status in order to maintain health as well as prevent accidents due to associated reduced cognitive capabilities. One of the objectives of this study was to document the hydration status of workers throughout the 12 hr work duration.

### Physical Fatigue

Intense or prolonged physical activity especially in the heat may result in fatigue. Though the causes, symptoms and performance consequences of fatigue are complex and variable, physical fatigue can be classified as either local or systemic. Local fatigue develops when the blood flow to a working muscle is inadequate, resulting in a reduced O_2 _supply and metabolite clearance. As O_2 _levels drop, the tissue relies increasingly on anaerobic metabolism with the production of lactic acid. Increased acidity and the accumulation of metabolites reduce the efficiency of energy production, limiting the work duration of the tissue. Local fatigue normally occurs in static or high intensity work. However light to moderate, long duration work is more commonly associated with systemic, or whole body fatigue. Systemic fatigue can be quantified by measuring the heart rate, O_2 _uptake, blood pressure, respiration rate, core body temperature, or perceived fatigue of a worker. Continuous heart rate recording is the most practical and informative measure, as it provides information about the total, peak and specific muscle work loads, the thermal stress of the environment, the work-rest pattern and the work pace or mental stress associated with the occupation [18].

Heart rates can be used to provide guidelines for acceptable work intensities. The World Health Organisation (WHO) has recommended that an average heart rate over the duration of a working shift should not exceed 110 beats min^-1^. This is somewhat below research findings that suggest performance deteriorates when mean working heart rates exceed 120 beats min^-1^[19]. An individual's maximum heart rate can be approximated by subtracting their age from 220 beats.min^-1^. Though the physiological basis for such guidelines is scant, ISO9886 advises that a person's heart rate should never exceed their maximum heart rate minus 20 beats.min^-1^[20].

A useful measure calculated from heart rates is the cardiac reserve, being the difference between the maximum and basal heart rates of an individual. When mean working heart rate is presented as a percentage of the cardiac reserve, this gives an indication of the sustainability of the workload being carried out. Percentage of cardiac reserve is approximately equivalent to % VO_2max_, or maximum oxygen uptake [21]. Increments in work intensity will increase heart rate and oxygen uptake (VO_2_) proportionally and therefore % cardiac reserve and % VO_2max_. Several studies have shown that a given work load is sustainable if % VO_2max _doesn't exceed 33–35% [22,23]. Core body temperature begins to rise if the % VO_2max _exceeds about 50%. The type of exercise being performed also influences VO_2max_. Upper body exercise is more demanding on the cardiovascular system than lower body work, consequentially the VO_2max _during arm work is about 70% that of work performed by the legs [24].

### Central Fatigue

Central fatigue refers to reduced central nervous system performance, experienced as mental tiredness or exhaustion. In cases where physical and mental fatigue occur simultaneously, there is often a perceived increment in the level of exertion required to complete a given task. Central fatigue however, often occurs without physical fatigue, particularly in occupations that are mentally or perceptually demanding [6].

Lack of sleep is a common cause of central fatigue. Performance decrements due to sleep loss are greatest in long duration tasks that are mentally demanding. Reduced CNS arousal in mentally fatigued subjects has been illustrated using EEG, which shows diminished electrical activity in the brain in response to auditory signals. Fatigue due to lack of sleep can also cause prolonged heart rate recovery periods after exertion, and increased resting heart rates. There is also a higher prevalence of sleep deprivation in night-shift workers [6].

Fatigue can be considered in a broader sense to encompass the lifestyle, health and welfare implications of working in a stressful or taxing environment. Industrial workers away from family and friends in the UAE present a myriad of psychosocial issues that may affect not only the workers, but also their spouse and families. Separation from partners and children may exacerbate fatigue.

The work-centered lifestyle and minimal leisure time of these workers means they have little time for recreational activities and exercise. Other health risk behaviours such as smoking and a poor diet may also present long-term implications for the health of these workers.

### Assessment of the Physical Environment

Physical labour in a hot and humid environment imposes considerable physical strain on the workers, with significant associated health risks. In order to maximise productivity without compromising a duty of care to employees, industrial operations in hot climates must carry out quantitative heat stress assessment of the workplace.

The degree of thermal stress imposed by a given environment depends upon a number of variables. These are the 'dry bulb' temperature, 'wet bulb' temperature (measuring humidity), wind speed (convection) and radiant heat. However, calculation of a threshold for 'safe' versus 'unsafe' work also requires consideration of factors affecting the individual worker. The work intensity, clothing worn, and the heat tolerance of the subject will all affect the risk of heat related illness or injury.

Several indices have been developed in an attempt to quantify thermal strain. A widely used index has been the Wet Bulb Globe Temperature (WBGT), which is still the standard in many industries. It has been used by the National Institute for Occupational Safety and Health (NIOSH) and the International Organisation for Standardisation (ISO) to set work limits and guidelines for work/rest cycling in thermally excessive environments. Calculated using the natural wet bulb, dry bulb and globe temperatures, the WBGT is compared to estimated metabolic work loads for the task or tasks being performed. From this it is established whether the environment is excessive given the required workload. The WBGT is relatively easy to measure and the instrumentation is not overly expensive, however it has several shortcomings as a measure of thermal stress. It does not incorporate direct measure of wind speed, and requires estimation of metabolic rates, which can have a margin of error up to 50% [25]. The guidelines are also unrealistic, as stringent application of the protocol would demand shutdown of virtually every construction site in the UAE during summer.

Recently developed indices have addressed the inadequacies of the WBGT to provide more meaningful and useful measures of environmental heat stress. Of these the most practical and informative is the Thermal Work Limit (TWL) [26], developed from published studies of human heat transfer and established heat and moisture transfer equations through clothing. The TWL is an integrated measure of the dry bulb, wet bulb, wind speed and radiant heat. From these variables, and taking into consideration the type of clothing worn and acclimatisation state of the worker, the TWL predicts the maximum level of work that can be carried out in a given environment, without workers exceeding a safe core body temperature and sweat rate. In excessively hot conditions, the index can also determine the safe work duration, thus providing guidelines for work/rest cycling. Sweat rates are also calculated, so the level of fluid replacement necessary to avoid dehydration can be established. The TWL guidelines have been implemented in several Australian mines, and have produced a substantial and sustained decrease in the number of cases of heat related illness. Measured in Watts.m^-2^, the TWL can also be used to calculate loss of productivity due to thermal stress and compare the cost of interventions (refrigeration, ventilation) with the decrement in productivity [26]. The current study used TWL as a thermal stress index during the working 12-hour day, whilst also computing WBGT for comparison.

## Methods

This study was carried out at a building construction site in Al Ain, an inland city in the United Arab Emirates, during May (approaching the summer months).

All participants were volunteers who gave their written and informed consent to participate in the study, which was authorised by management and approved by the Al-Ain Medical District Human Research Ethics Committee.

At the commencement of the study general demographic, health-risk behaviours, and lifestyle data was obtained by interview, as was anthropometric data in the form of height, weight, and BMI for each individual worker.

A total of 22 subjects (divided into 3 groups) were studied, each group over 3 consecutive days (a total of 66 subject/day records over 9 study days). The first group was comprised of carpenters, the second steel fixers, and the third general labourers. All workers were male expatriates working 12-hour shifts, 6 days per week. All were employed by a labour hire company, and were provided with air-conditioned sleeping quarters at the labour camp. Twelve had been recruited from India and ten from Bangladesh.

The workers were engaged in the construction of a large concrete water feature outside of a multi-story office building. The nature of the work precluded any provision of shade other than that offered by the nearby building. An air-conditioned mess hall was used for the 1-hour meal break and ample supplies of cool water were readily available on site, and their consumption encouraged by the contractor.

The objectives of the study were:

• To determine if workers were becoming physically fatigued during the 12 hr shift and over a 3 day period, using heart rate monitoring

• To identify and assess any trends in the hydration status of workers over the shift duration and from day 1–3.

• To perform a workplace heat-stress risk assessment using the Thermal Work Limit as an index.

### Worker Monitoring

#### Fluid intake

Fluid consumption was determined by allocating a separate water container to each worker participating in the study. This personal water container was located in a central point and a record was kept of the number of times it required refilling. From this and the residual water left in the container at the end of the shift fluid consumption could be calculated. A record was also kept of additional fluid intake in the form of tea, coffee, or soft drinks consumed during the shift.

#### Hydration status

Hydration status was determined by measuring the specific gravity (SG) of urine samples collected from subjects at the start, middle, and completion of each shift. SG was measured using a handheld, calibrated, "Atago" optical urine refractometer.

#### Physiological strain

Volunteers were fitted with Polar S720i heart rate monitors, which supplied continuous HR data (1 recording every 30 sec). The data was downloaded at the end of each shift and the data used to calculate mean and maximum working heart rates as well as percentage of cardiac reserve. Resting heart rates were taken while the subject was at rest before the start of the first shift. The participants each wore the monitors for 3 consecutive days. Average heart rates for the morning and afternoon sections of the shift were calculated to identify physical fatigue developing through the shift.

Core body temperature measurement was also recorded at the beginning and end of each shift using tympanic thermometers with disposable probe shields, which were discarded after each use.

### Workplace monitoring of environmental conditions

In order to quantify the level of environmental heat stress, the environmental conditions were monitored at the workplace on 4 occasions (9 am, 12 md, 2 pm and 4 pm) during each shift. A Calor Heat Stress meter was used to determine wet (WB) and dry bulb temperature (DB), black globe temperature (radiant heat), wind speed, and barometric pressure and from these measurements calculations of mean radiant temperature, relative humidity, WBGT and Thermal work limit (TWL) values were determined.

### Statistics

Pearson's correlation was performed on all data sets.

## Results

Table 1 summarises the average results over all groups for each of the three days (1–3) of the study; Pearson correlation coefficients between fluid consumption and both urine SG and working heart rates are given in table 2.

**Table 1:** Average total fluid consumption, urine SG and working heart rate for each day of the study

**Average**	**Day 1**	**Day 2**	**Day 3**
Fluid consumption (mL)	6001 ± 1396	5235 ± 1388	5044 ± 1133
Urine SG (mean of three samples per day)	1.011 ± 0.008	1.013 ± 0.007	1.013 ± 0.006
Heart rate (beats.min^-1^)	90.5 ± 8.1	90.0 ± 5.9	86.9 ± 6.5

Values are mean ± SD, n = 22 subjects

**Table 2:** Correlations between individual fluid consumption and average urine SG and heart rate

**Variables (n = 22)**	**Pearson correlation coefficient**
Average fluid consumed Average SG for 3 days	-0.519*
Average fluid consumed for 3 days Average heart rate over 3 days	0.719**

*Significant at the 0.05 level (2-tailed)

**Significant at the 0.01 level (2-tailed)

**Figure 1:**
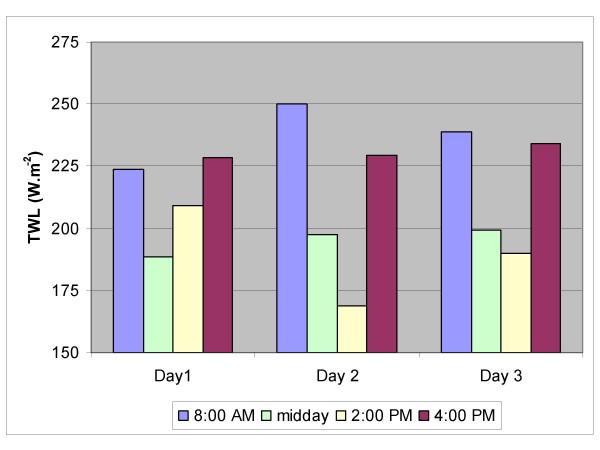
**Thermal Work Limit (TWL)**. The Thermal Work Limit was recorded on four occasions per day, and averaged for each of the three study days.

**Figure 2:**
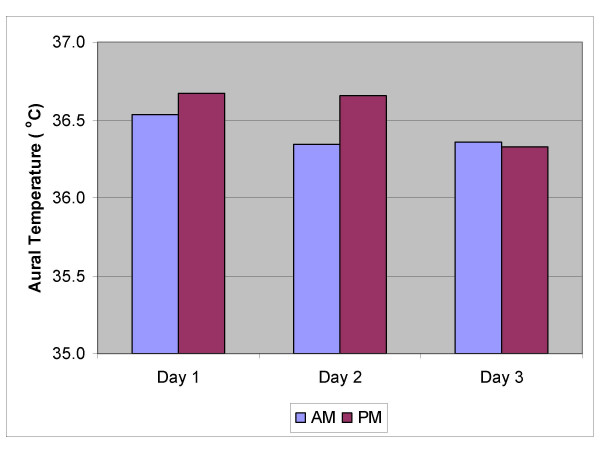
**Aural Temperature am & pm**. Core temperature was monitored by measurement of aural temperature twice daily. Averages for each day of the study are shown.

**Figure 3:**
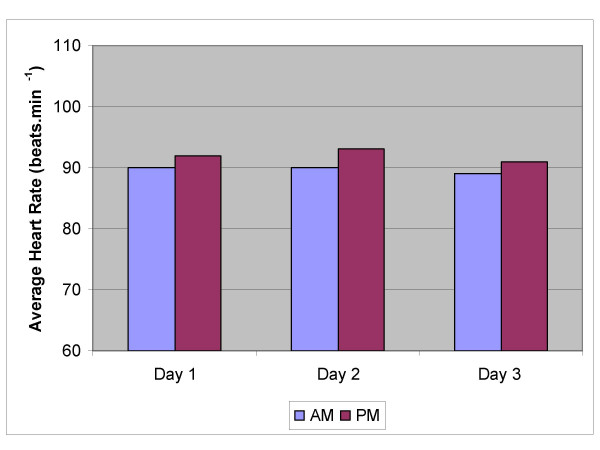
**Average Heart Rates**. Averages of the continuously recorded heart rates for the morning and afternoon work period of each of the three study days.

**Figure 4:**
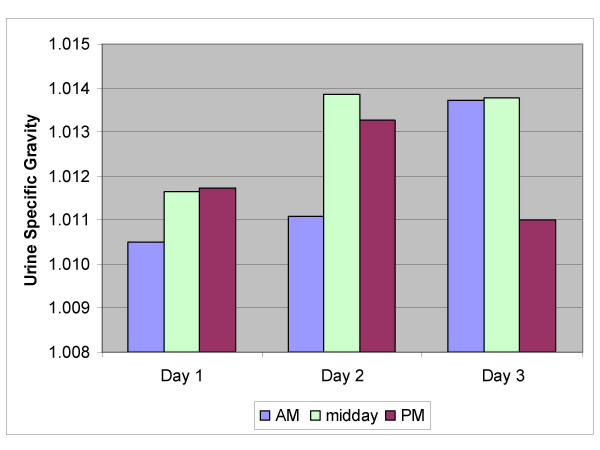
**Urine Specific Gravity**. Average specific gravity of urine measured at the start and end of shift and during the lunch break.

**Figure 5:**
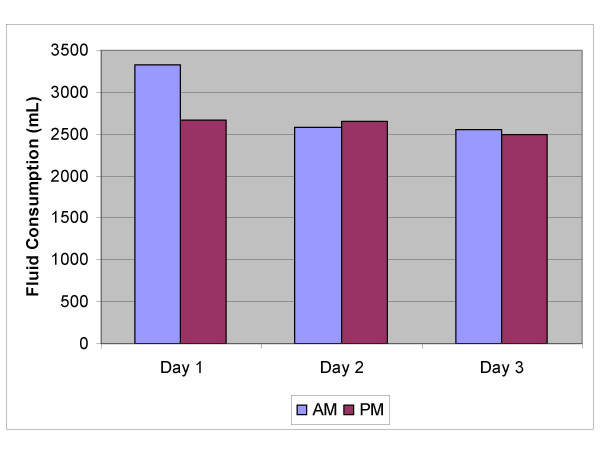
**Fluid Consumption**. Volume of fluid consumed by workers during the morning and afternoon for each of the three study days.

Figures 1, 2, 3, 4, 5 show the breakdown by time of day for subject variables and environmental conditions.

The environmental conditions were recorded on four occasions per day. Table 3 shows mean and range for each parameter over the nine days of the study and the WBGT and TWL values computed from these. The environmental stress as measured using the TWL, altered considerably over the duration of the day (fig 1). The stress was lower in the morning and late afternoon readings; whilst at midday it was harsher as indicated by the lower TWL readings on all 3 days. Despite this there were no significant differences in subject variables either within or between days, and in fact TWL rarely fell below the limit for performance of unrestricted work by self-paced workers (table 4). In comparison WBGT values consistently exceeded 27.5°C, the recommended limit for moderate work, especially during the middle of the day [27].

**Table 3:** Environmental conditions over the study period

**Time**	**DB ** **(°C)**	**WB** **(°C)**	**GT** **(°C)**	**WS ** **m.s^-1^**	**WBGT** **(°C)**	**TWL ** **W.m**^-2^
**0800**	37.9 (32.5–44.0)	21.3 (19.4–24.3)	44.8 (38.5–51.2)	1.4 (0.4–2.0)	26.8 (24–30.7)	237.7 (179–284)
**1200**	42.5 (40.1–48.2)	21.8 (18.4–24.9)	52.1 (56.5–49.2)	1.7 (0.8–3.1)	28.6 (26.9–30.8)	194.8 (151–225)
**1400**	44.7 (42.7–49)	20.6 (17.3–23.2)	51.8 (47.7–55.5)	2.0 (1.3–4.6)	27.8 (26.9–28.9)	189.3 (122–240)
**1600**	41.0 (32.9–46.6)	19.0 (16.4–22.3)	44.3 (33.9–53.1)	2.4 (0.3–6.2)	26.1 (24.5–27.9)	230.6 (187–279)

DB = dry bulb, WB = wet bulb, GT = globe temperature (radiant heat), WS = wind speed, WBGT = Wet Bulb Globe Temperature, TWL = Thermal Work Limit

Values are mean (n = 9) and range (parentheses)

**Table 4:** Recommended TWL limits and interventions for self-paced work

**TWL Limit (W.m^-2^)**	**Name of limit/zone**	**Interventions**
< 115	Withdrawal	No ordinary work allowed
		Work only allowed in a safety emergency or to rectify environmental conditions

115 to 140	Buffer zone	Try to improve the working environment
		No person to work alone
		No unacclimatized person to work

> 140	Unrestricted	

Figure 2 shows that the aural temperatures of the workers (n = 22) were constant over the 3 days of the study, and as shown in figure 3, heart rates did not alter significantly throughout the shift or from day to day, despite a significant increase in environmental thermal stress, suggesting that the workers were not being physically fatigued during their shift.

The hydration data (fig 4) demonstrate that the workers commenced work well hydrated and maintained their hydration status throughout the shift and from day 1 to day 3 (n = 66).

The average fluid intake of workers (n = 22) was reasonably consistent during the day and from day 1-day 3 (fig 5).

**Figure 6:**
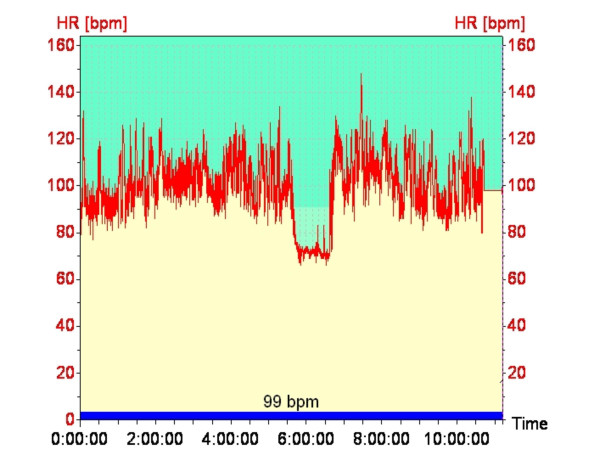
**Typical Heart Rate Recording**. Continuous heart rate recording over a full shift, from one of the workers. The lunchtime meal break is clearly evident.

The constancy of working heart rate throughout the shift and the absence of environmental influence is demonstrated in (fig 6), a typical recording over a full shift, from one of the workers. The lunchtime meal break is clearly evident.

## Discussion

### Hydration

Maintaining body fluid levels whist working in a hot environment is essential, not only for health and safety of the worker, but in order to optimise performance and productivity.

Urine specific gravity is a measure of urine osmolarity and is related to the hydration status of the subject. It is recognized that false negatives can occur in persons consuming large volumes of caffeinated beverages, however, it is a very useful indicator for worksite screening of the hydration status of workers. Low readings are indicative of appropriate fluid levels in the body. From previous work on the hydration status of workers exposed to heat, a urine SG below 1.020 at the commencement of a shift is optimal to prevent hypohydration or dehydration further into the shift. It has been reported that workers are unlikely to improve their hydration status during work [2]. Thus it is imperative that good hydration prior to the shift commencement is achieved. The results of this study have illustrated very good hydration prior to the commencement of the shift, which is also maintained over the course of the shift. Workers who begin well hydrated are likely to maintain good levels of hydration during the shift. Indeed, most participants in this study commenced work in a euhydrated state, the average SG over the 3 days being 1.012 (fig 4).

This highlights the need for an active education program promoting awareness about the importance of hydration and offering practical advice to workers. Key components of such a program would be discussion of the health, safety and performance implications of adequate hydration, as well as information regarding what, when and how much to drink. The average intake of hydrating fluids per 12-hour shift was 5.44 litres (fig 5), which was adequate, as SGs were maintained during the shift. Furthermore, the type and calorific content of any hydrating fluid needs consideration, given that juice, cordial and other sweet beverages are often more than 10% sugar. Caffeinated beverages such as tea, coffee, cola and energy drinks may dehydrate rather than hydrate workers. Another factor that may have significant bearing on the hydration status of these workers is cultural. Most reported no alcohol consumption due to their religious beliefs. Maintenance of an adequate hydration level maybe learnt, becoming in effect a physiological 'set point', as some workers sustained consistently lower SGs than others. (Interpretation of urine specific gravity and associated hydration levels is provided in table 5)

**Table 5:** Guidelines for interpretation of urine Specific Gravity readings

**SG**	**Significance**
< 1.015	Well Hydrated
1.015–1.020	Mildly Dehydrated
1.020–1.025	Moderately Dehydrated
1.025–1.030	Dehydrated
> 1.030	Clinically Dehydrated

### Fatigue

Fatigue is a complex process with physiological, psychological and sociological components and implications. A major consequence of any type of fatigue is reduced productivity due to diminished work efficiency. Fatigue also increases the likelihood of workplace errors and accidents, and as a consequence, is a significant concern in industrial operations such as the construction and oil industry.

The primary objective of this study was to assess the physiological stress associated with working for long periods in a hot environment. The continuous heart rate monitoring demonstrated no significant change in heart rate between the morning and afternoon shift periods or from day 1 to day 3, suggesting that workers are not fatiguing over the duration of a shift (am vs pm) or from day to day (fig 3). There may be two possible explanations for this; either workers are not becoming fatigued, or they are self-pacing, that is, slowing down to avoid over-exertion. The latter seems most likely, and would appear to be the key factor in avoiding heat related injury. Other work has shown similar results [28]. The environment (thermal stress) changes significantly over the course of the day (fig 1), however heart rates remain constant over the day and from day to day. It is not fanciful to suggest that workers if allowed to self-pace will alter work rate to maintain their heart rate within a narrow range. These workers varied in fitness level and experience; however they all worked at a similar heart rate. It is recognized that the number of subjects (n = 22) is not sufficient to conclude that workers even in harsh conditions (DB temperature reached 53°C on one occasion and was reaching the mid to high 40's most days) will be safe if they are well hydrated and allowed to self-pace, however it is good evidence for promoting a more rigorous study using a far greater number of workers.

The value of these findings may alter the current approach to working in heat, which is to stop work when a single environmental parameter reaches a threshold point or the cessation of work during the hottest part of the day during summer. These guidelines and legislative regimes are unscientific and often cause more problems than they solve (industrial disputes, as well as unnecessary production costs and delays).

The relationship between heart rate and fluid consumed (table 2) was positive (correlation coefficient 0.719). One likely explanation was that those workers who worked harder (higher heart rates) drank more fluid. An alternate explanation may be that those that drink more fluid can work harder. The latter explanation, if correct, would be of significant interest to employers and may promote better supply and availability of suitable fluid on work sites.

The other significant correlation was between SG of urine and average fluid consumed (table 2). As would be expected those that drank more fluid had a lower SG thus an inverse relationship (Pearson correlation -0.519). This would endorse the validity of using SG as an indicator of hydration. No other statistically significant correlations were recorded.

### Environmental Assessment

A risk assessment of the thermal environment at the construction site was carried out over a 10-day period during the month of June, using the Thermal Work Limit (TWL) as a measure of heat stress. The workplace was assessed on 4 occasions daily to identify variation in thermal stress. Though the average TWL for most work sites was above the stop work level, i.e. above 115 W.m^-2 ^(table 4), on occasions the risk of heat strain in certain working environments did become substantial, reaching TWL levels as low as120 W.m^-2 ^(DB temp > 50°C) however this was not reflected in the heart rates for that specific time nor the reporting of symptoms or deleterious effects on the workers. By comparison there were few days during the study when risk assessment using WBGT would not have required work to be shut down for at least part of the day. This reinforces the proposition that self-pacing in the construction industry is imperative if heat illness is to be avoided. The other important point illustrated by this data is the importance of good hydration of the workforce.

## Conclusion

The data demonstrate that well hydrated self-paced workers can work without adverse physiological effects under conditions deemed too severe by the WBGT. It is now recognized that WBGT is too conservative and inappropriate for practical use in industry. A more scientifically robust index is urgently needed, especially in the hotter parts of the globe where workers are performing manual tasks in very harsh conditions. The debate as to what is a reasonable environment in which people work, will become a more and more pertinent question. A far greater push to establish an index that will both protect workers yet not punish industrial productivity is well overdue. TWL has been published and validated in a controlled environment [28,29]. Introducing TWL as a practical measure of heat stress in industrial settings where heat is an issue would appear to be appropriate. It measures all needed environmental parameters, takes into account clothing and provides the metabolic rate (the output) that people can sustain in a specific environment (in W.m^-2^).

Additional physiological testing of workers along with environmental measurements need to be conducted in order to further validate the recommended levels shown in table 4, however to date the field testing undertaken in this study and in the laboratory validation studies provide very good evidence for it to be taken seriously as a international index that can be relied upon to be a sound independent arbitrator for people working in harsh thermal environments.

## Competing interests

The authors declare that they have no competing interests.

## Authors' contributions

JS conceived the study, which was designed by GB. Both authors collected data. GB analysed the data and interpreted the results. Both authors drafted, edited and approved the final manuscript.
